# Synergistic Effects of Pulsed Lavage and Antimicrobial Therapy Against *Staphylococcus aureus* Biofilms in an *in-vitro* Model

**DOI:** 10.3389/fmed.2020.00527

**Published:** 2020-09-17

**Authors:** Hervé Poilvache, Albert Ruiz-Sorribas, George Sakoulas, Hector Rodriguez-Villalobos, Olivier Cornu, Françoise Van Bambeke

**Affiliations:** ^1^Laboratoire de Neuro-Musculo-Squelettique, Institut de Recherche Expérimentale et Clinique, Université Catholique de Louvain, Brussels, Belgium; ^2^Laboratoire de Pharmacologie Cellulaire et Moléculaire, Louvain Drug Research Institute, Université Catholique de Louvain, Brussels, Belgium; ^3^Orthopaedic Surgery Department, Cliniques Universitaires Saint-Luc, Brussels, Belgium; ^4^School of Medicine, University of California, San Diego, San Diego, CA, United States; ^5^Clinical Microbiology Department, Cliniques Universitaires Saint-Luc, Brussels, Belgium

**Keywords:** biofilm, MRSA, MSSA, pulsed lavage, vancomycin, flucloxacillin, prosthetic joint infection

## Abstract

**Background:** Prosthetic joint infections (PJI) are difficult to treat complications of joint arthroplasty. Debridement with implant retention is a common treatment strategy and frequently involves the use of pulsed lavage (PL). However, PL effects on biofilms and antibiotic activity have been scarcely studied *in-vitro*. We report the effects of PL, vancomycin or flucloxacillin used independently or in combination against *Staphylococcus aureus* biofilms.

**Methods:** Biofilms of 3 methicillin-susceptible (MSSA) and of 3 methicillin-resistant (MRSA) *S. aureus* were grown on Ti6Al4V coupons in TGN (TSB + 1%glucose + 2%NaCl). After 24 h, PL was applied to half of the samples (50 mL saline from 5 cm). Samples were either reincubated for 24 h in TGN or TGN + flucloxacillin or vancomycin. Analyses included CFUs counts, biomass assays or fluorescence microscopy.

**Results:** PL transiently reduced bacterial counts by 3–4 Log_10_ CFU/coupon, but bacterial regrowth to baseline levels was seen after 24 h. At 20 mg/L, flucloxacillin reduced both the CFU counts (3 Log_10_ CFU/coupon) and biomass (−70%) in one MSSA only, while vancomycin had no effects against MRSA. PL combined with a 24 h reincubation with vancomycin or flucloxacillin at 20 mg/L was synergistic (−5 to 6.5 Log_10_ CFU/coupon; 81–100% biomass reduction). Fluorescence microscopy confirmed that PL removed most of the biofilm and that subsequent antibiotic treatment partially killed bacteria.

**Conclusions:** While PL only transiently reduces the bacterial load and antibiotics at clinically relevant concentrations show no or limited activity on biofilms, their combination is synergistic against MRSA and MSSA biofilms. These results highlight the need for thorough PL before antibiotic administration in PJI.

## Introduction

Prosthetic Joint Infections (PJI), defined as infections involving joint replacement implants and the surrounding articular tissues, are devastating complications, affecting 0.5 to 2% of patients benefiting from hip or knee replacement (1, 2) and are among the most common causes of arthroplasty failures (3, 4).

These infections result from either a peri-operative contamination of the joint, generating acute (less than 4 weeks from the index surgery) or late infections, or an hematogenous seeding of bacteria to the joint following a bacteremia (2). The prevalence of the causative micro-organisms varies depending on the origin and interval from the index surgery, with *Staphylococcus aureus* being the most frequently isolated in cases of acute PJI, whereas coagulase-negative staphylococci and *Streptococci* spp. predominate late infections and hematogenous infections, respectively (5–8).

Infections by *S. aureus* are characterized by the rapid adhesion of bacterial cells to the implant surface, followed by the development of a self-produced extracellular matrix composed of poly-*N*-acetylglucosamine, extracellular DNA, proteins, and lipids, forming complex communities known as biofilms (6). The development of the biofilm induces phenotypic changes of the bacteria, which, combined with the isolating effects of the matrix, make bacteria tolerant to antibiotics at up to 1000x the minimal inhibitory concentration (MIC) observed in a planktonic state (9). This explains the limited success of antimicrobial therapy and the necessity for surgical strategies aiming to disrupt or remove the biofilm (2).

The Debridement, Antibiotics and Implant Retention (DAIR) strategy is often recommended for the treatment of acute PJI due to lower morbidity and costs than staged implant replacement. The surgical procedure consists in the open debridement of the infected joint, with the excision of necrotic tissues and a synovectomy, replacement of bearing surfaces if possible, followed by a thorough lavage of the joint space usually performed with a pulsed-lavage device which projects normal saline intermittently at pressures between 30 and 350 kPa (10, 11). However, DAIR presents a relatively high failure rate (16–57.4%), with a worse prognosis for patients infected with *S. aureus* (12–16). These failures may be partly explained by an inadequate removal of biofilms during the debridement surgery and their tolerance to antibiotics. However, only a few studies have looked into the effects of irrigation performed using standard pulsed lavage devices against *S. aureus* biofilms grown on metallic substrates, and none of these investigated the effects of its combination with antibiotics at clinically relevant dosages for systemic administration (17–20). The purpose of this study was to describe the effects of pulsed lavage and clinically relevant antibiotics used at recommended concentrations for systemic use, (i) in combination or (ii) independently, on the amount of cultivable cells, the biomass, and the microscopic aspects of MRSA and methicillin-susceptible *S. aureus* (MSSA) biofilms on titanium alloy coupons.

## Materials and Methods

### Bacterial Strains

The laboratory strains ATCC 25923 and ATCC 33591 were used as references for MSSA and MRSA biofilms, respectively. Two MSSA clinical isolates (strains 578 and 611) and two MRSA clinical isolates (strains 676 and 749), collected from orthopedic device-related infections cases were also studied.

### Antibiotics

Oxacillin (powder potency: 81.5%) was obtained as a microbiological standard from Sigma-Aldrich (Sigma-Aldrich Corp., Saint-Louis, MO, USA). Vancomycin (Vancomycin Mylan, powder potency: 97.5%, Mylan Inc, Canonsburg, PA, USA) and flucloxacillin (Floxapen, powder potency: 91.9%, Actavis Group, Hafnarfjördur, Iceland) were used as a powder for injection approved for human use in Belgium.

### Susceptibility Testing

MICs were determined by broth microdilution in cation-adjusted Mueller-Hinton broth (CA-MHB, Sigma-Aldrich Corp., Saint-Louis, MO, USA) as per the Clinical & Laboratory Standards Institute protocol (21), and in Tryptic soy broth (VWR Chemicals, Leuven, Belgium) supplemented with 1% glucose (Sigma-Aldrich Co., Saint-Louis, MO, USA) and 2% NaCl (VWR Chemicals, Leuven, Belgium) (TGN) (Table 1).

**Table 1:** MIC (mg/L) values for the tested strains^a^.

**Strains**		**Oxacillin^b^**	**Flucloxacillin**		**Vancomycin**	
		**CA-MHB**	**CA-MHB**	**TGN**	**CA-MHB**	**TGN**
MSSA	ATCC 25923	0.25	0.13	0.06	1	8
	578	0.25	0.25	0.13	2	8
	611	0.25	0.25	0.06	1	8
MRSA	ATCC 33591	>64	>64	>64	1	4
	749	>64	>64	>64	1	8
	676	>64	64	64	1	8

a *CLSI breakpoints values (in CA-MHB; mg/L): Flucloxacillin: N/A; Oxacillin: S ≤ 2, R≥4; Vancomycin: S ≤ 2, R≥16*.

b *used to check the MRSA character of the strain, according to CLSI guidelines (21)*.

### Biofilm Culture

Biofilms were grown on titanium alloy Ti6Al4V coupons (Biosurface Inc., Bozeman, MT, USA) in order to mimic implant surface characteristics. These coupons are unpolished cylinders measuring 12.7 mm in diameter and 3.175 mm in height. The initial inoculum was prepared from bacteria grown overnight on Tryptic Soy Agar (VWR, Leuven, Belgium) (TSA), suspended in Phosphate Buffer Saline (PBS), adjusted to an optical density at 620 nm of 0.5 (CECIL 2021 spectrophotometer, CECIL, United-Kingdom) and diluted 1:100 in TGN, reaching a bacterial density of ~6.5 log_10_ CFU/mL. Sterile coupons were incubated for 24 h at 37°C in 12 wells plates containing 2mL of bacterial suspension in TGN per well, under a continuous orbital shaking of 50 rpm in order to induce shear stress. Biofilms reached maturity after 24 h (i.e., no meaningful change in biomass or bacterial counts was observed when prolonging the incubation for 48 h, Supplementary Data Figure 1) and used for testing the treatments.

### Biofilm Treatments

#### Irrigation

Half of the biofilm samples (referred to as irrigation samples) were irrigated with 50 mL of sterile saline (Baxter International Inc, Deerfield, IL, USA) from 5 cm, using Interpulse battery-powered irrigation devices (Stryker Co., Kalamazoo, MI, USA). The Interpulse were fitted with soft tissue tips, delivering the sterile saline at a flow rate of 700 ml/min and a pressure of 68.95–82.74 kPa or 10–12 PSI (manufacturer's data). The samples were then rinsed twice in sterile PBS before allocation to one of the subgroups. The other half of the samples (referred to as control samples) were rinsed twice in sterile PBS before being allocated to one of the subgroups.

#### Antibiotic Treatments

**Figure 1:**
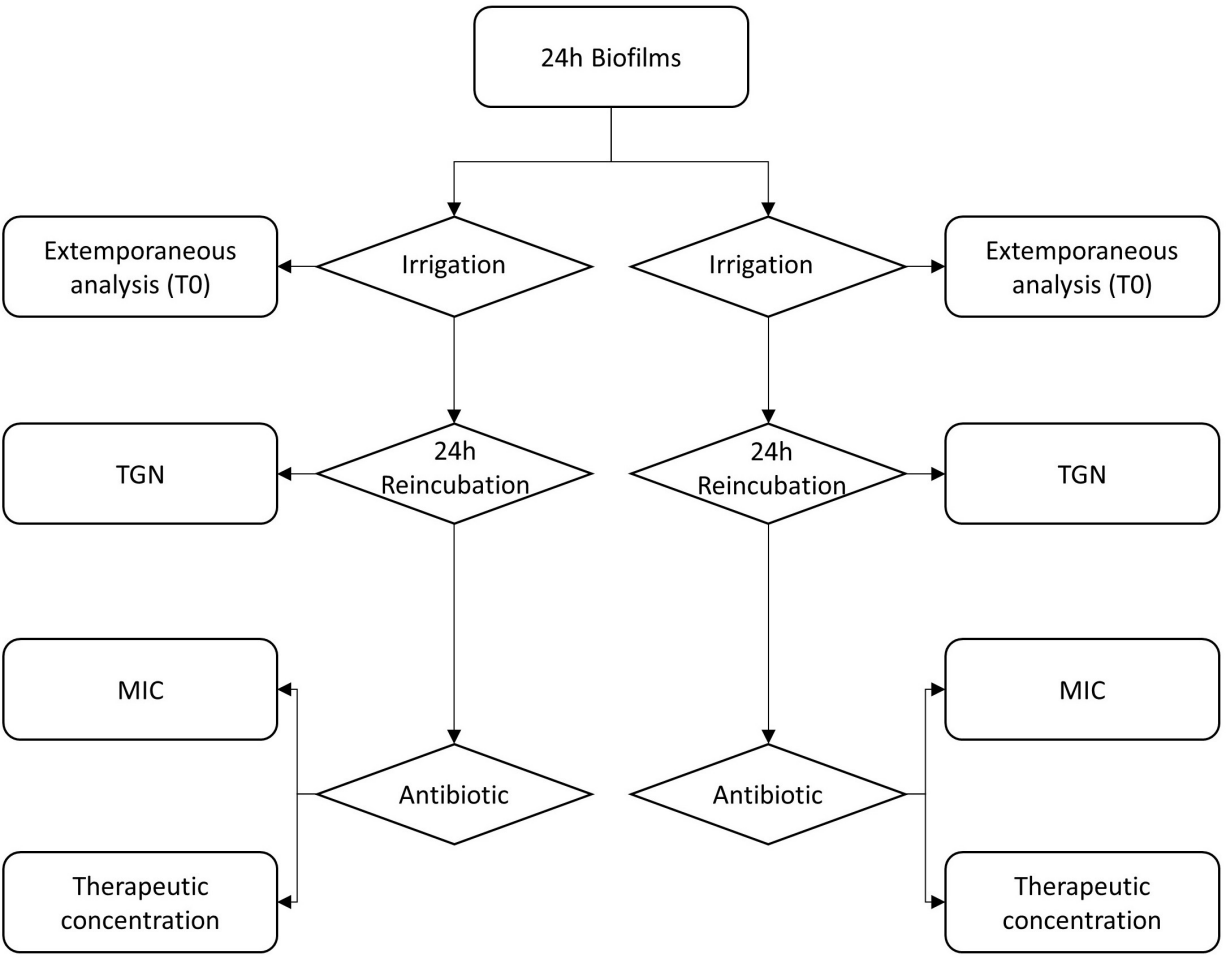
Flowchart describing the experimental design.

Control and irrigation coupons were allocated to one of the subgroups: immediate analysis (T0); 24 h reincubation in TGN (T24h—TGN); 24 h reincubation in TGN containing antibiotic at MIC (T24h–MIC); 24 h reincubation in TGN containing therapeutic concentration of antibiotic (T24h–ThC) according to the flowchart shown in Figure 1. Reincubations were done at 37°C, under a continuous rotating movement at 50 rpm. As antibiotics, flucloxacillin was used for MSSA biofilms, considering as therapeutic concentration 20 mg/L, an estimate of the serum concentration 3 h after injection when administered 2 g IV four times daily, as inferred from pharmacokinetic data (22). MRSA biofilms were reincubated with vancomycin at a therapeutic concentration of 20 mg/L, corresponding to target trough serum concentration for bone and joint infections (23).

### Biofilm Analysis

#### CFU Counts

Coupons were individually placed in 15 mL conical tubes (Greiner Bio-One International GmbH, Kremsmünster, Austria) containing 2 mL of sterile PBS. The tubes were vortexed for 30 s, sonicated for 5 min (Branson 5510 Ultrasonic bath, Emerson Electric, Saint-Louis, MO, USA) and vortexed 30 s again. Aliquots of the supernatant were serially diluted, plated on TSA and incubated for 24 h at 37°C. CFU counts were performed using an automated method [image acquisition using Gel Doc XR+ and image processing using Quantity One (BioRad, Hercules, CA, USA)].

#### Biomass Quantification

After drying overnight at 60°C, coupons were stained with 1 mL of 1% crystal violet (Sigma-Aldrich Corp., Saint-Louis, MO, USA). After eliminating the excess of dye by rinsing the samples with deionized water, biofilm-bound crystal violet was resolubilized in 1 mL of a 66% acetic acid (Merck KGaA, Darmstadt, Germany). The coupons were then removed from the solution and the absorbance was read at 570 nm using a Spectramax M3 spectrophotometer (Molecular Devices, San Jose, CA, USA).

#### Fluorescence Microscopy

Samples were stained using the FilmTracer LIVE/DEAD biofilm viability kit (ThermoFisher, Waltham, MA, USA) following the manufacturer's instructions. DAKO fluorescence mounting medium (Agilent, Santa Clara, CA, USA) was added after staining and a coverslip was placed. Images were acquired as Z-stacks using an AxioImager.Z1 microscope fitted with an ApoTome1 attachment (Zeiss, Oberkochen, Germany) at a 20x magnification using the structured light illumination technique. Image post-processing was performed using FIJI (24, 25). Images were reconstructed using Maximum Intensity Projection (MIP) and were further post-processed by increasing the brightness of each channel separately to the maximum value.

### Statistical Analysis

CFU counts were transformed to logarithmic values before statistical analysis. Biomass values were normalized as the percentage of positive controls after subtracting the average value of negative controls (coupons incubated in sterile TGN). Statistical analysis was performed using the mean of each repetition (*n* = 4, with *n* = 3 per replicate) using GraphPad 7.01 (GraphPad Software, San Diego, CA, USA). Means were compared using 2-way ANOVA, followed by Holm-Sidàk *post-hoc* test. Differences were considered statistically significant when *p* <0.05. Synergy was defined as a significant interaction factor (26).

## Results

### Antimicrobial Susceptibility

Minimal inhibitory concentrations (MIC) for the MSSA and MRSA strains are shown in Table 1. MSSA strains exhibited low MICs to flucloxacillin in either media, with MICs in TGN one to two dilutions lower than in CA-MHB. MRSA strains were resistant to flucloxacillin in both media. All strains were susceptible to vancomycin in CA-MHB, but their MIC was 2 to 3 dilutions higher when tested in TGN.

### Antimicrobial Activity in Biofilms

**Figure 2:**
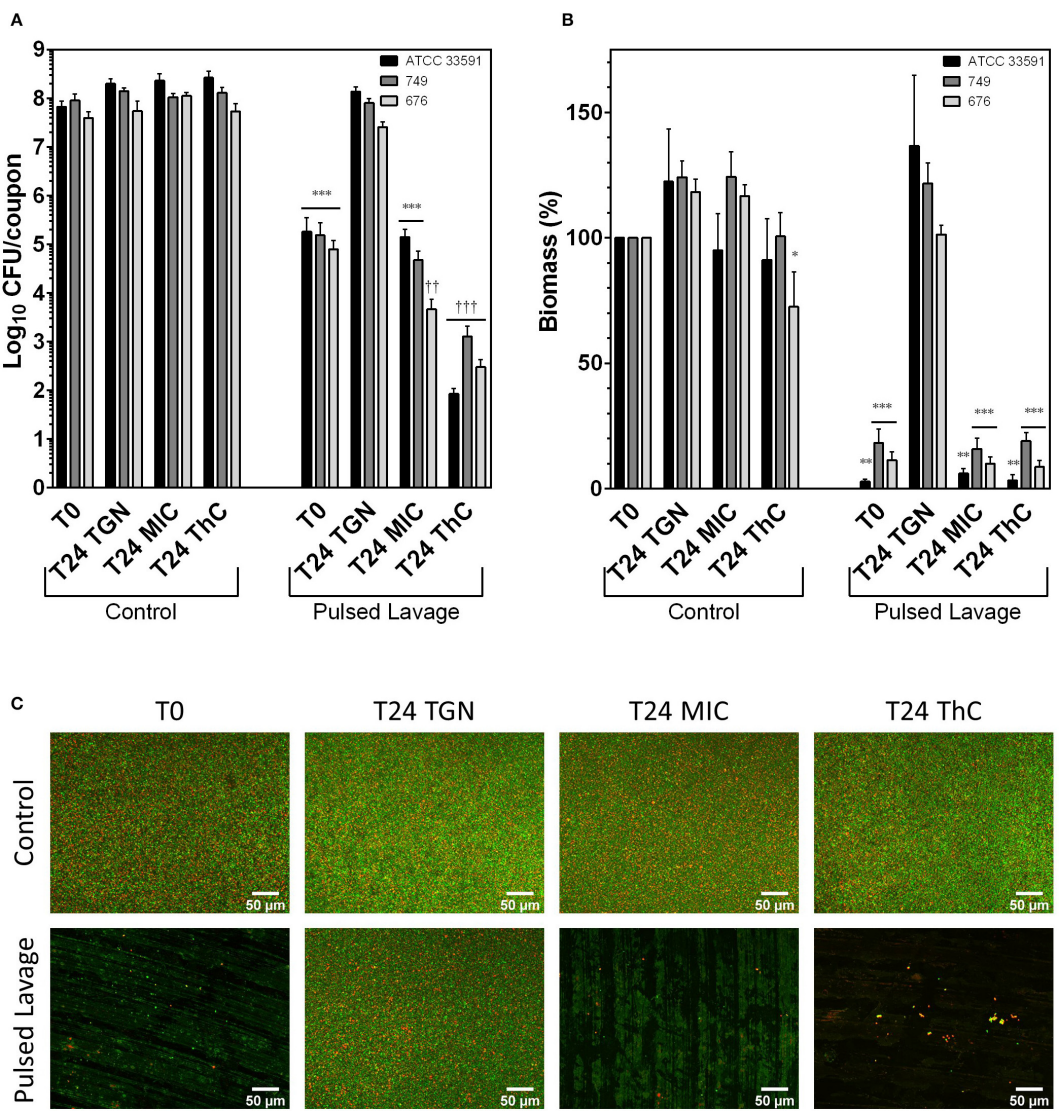
Effects of irrigation and vancomycin on MRSA biofilms grown for 24 h in TGN. **(A)** CFU counts; **(B)** biomass expressed in percentage of T0 controls; **(C)** MIP of Z-stack acquired at 20x magnification following Live (green fluorescence, Syto 9) /Dead (red fluorescence, Propidium Iodide) staining. Scale bar: 50 μm. Control: control groups; Pulsed Lavage; groups treated with pulsed lavage; T0: samples analyzed after 24 h of growth; T24 TGN: T0 samples analyzed after 24 h of reincubation in TGN; T24 MIC: samples analyzed after 24 h of reincubation in TGN with vancomycin at MIC; T24 ThC: samples analyzed after 24 h of reincubation in TGN with vancomycin at 20 mg/L (therapeutic concentration). Data expressed as means of experiments and SEM. N experiments ≥ 3. Statistical analysis: two-way ANOVA followed by Holm-Sidàk *post-hoc* test. Comparisons to T0 control samples: *p < 0.05; **p < 0.01; ***p < 0.001. Comparisons to T0 irrigation samples: ^†^*p* < 0.05; ^††^*p* < 0.01; ^†††^*p* < 0.001.

**Figure 3:**
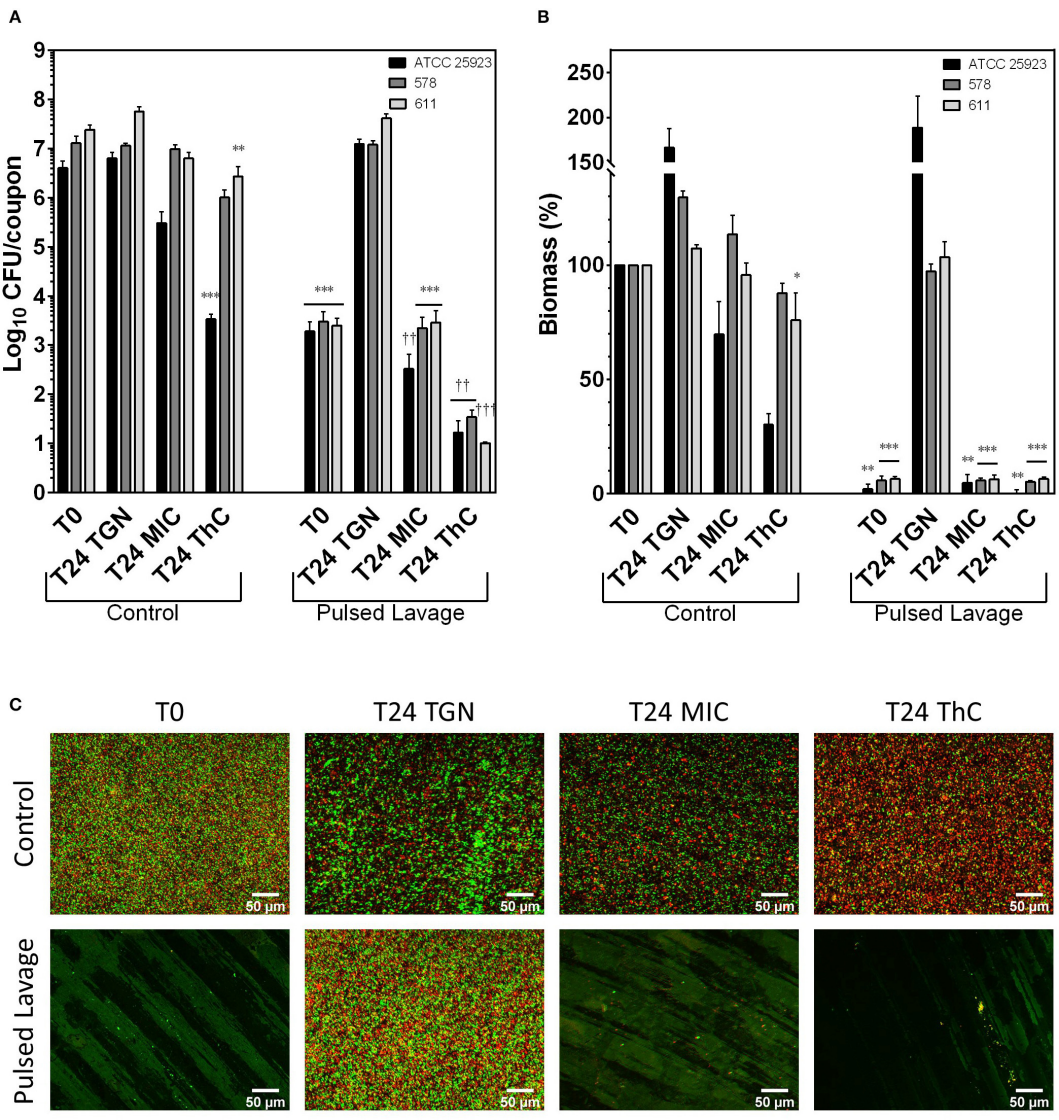
Effects of irrigation and flucloxacillin on MSSA biofilms grown for 24 h in TGN. **(A)** CFU counts; **(B)** biomass normalized as a percentage of T0 controls; **(C)** MIP of Z-stack acquired at 20x magnification following Live (green fluorescence, Syto 9) /Dead (red fluorescence, Propidium Iodide) staining. Scale bar: 50 μm. Control: control groups; Pulsed Lavage; groups treated with pulsed lavage; T0: samples analyzed after 24 h of growth; T24 TGN: T0 samples analyzed after 24 h of reincubation in TGN; T24 MIC: samples analyzed after 24 h of reincubation in TGN with flucloxacillin at MIC; T24 ThC: samples analyzed after 24 h of reincubation in TGN with flucloxacillin at 20 mg/L (therapeutic concentration). Data expressed as means of experiments and SEM. N experiments ≥ 3. Statistical analysis: two-way ANOVA followed by Holm-Sidàk *post-hoc* test. Comparisons to T0 control samples: *p < 0.05; **p < 0.01; ***p < 0.001. Comparisons to T0 irrigation samples: ^†^*p* < 0.05; ^††^*p* < 0.01; ^†††^*p* < 0.001.

Bacterial counts (CFU) are shown in Figure 2A for MRSA strains and in Figure 3A for MSSA strains. The CFU counts of control coupons of all strains did not change between T0 and T24, indicating biofilm maturity at T0. Incubation of control MRSA coupons with vancomycin at either MIC or 20 mg/L concentrations did not result in reductions in CFU counts when compared to the T0 control samples. A similar observation was made for MSSA biofilms exposed to flucloxacillin at MIC. However, exposure to flucloxacillin at 20 mg/L resulted in a statistically significant decrease of the CFU counts in strains ATCC 25923 (-2.98log_10_) and 611 (−1.49log_10_). The results of biomass assays are shown in Figure 2B for MRSA strains and Figure 3B for MSSA strains. As we observed with CFU counts, no statistically significant differences in biomass were observed between controls at T0 and T24, except for strain 578 (+29.6%, *p* < 0.001). Twenty-four hours exposure to vancomycin at MIC did not reduce biomass in control coupons of all MRSA strains. Exposure to a concentration of 20 mg/L did not affect the biomass, except for strain 676 (−27.5%, *p* = 0.03). The biomass of control MSSA samples was not modified in a statistically significant manner after a 24 h incubation with flucloxacillin at MIC. The diminutions of biomass of ATCC 25923 samples observed after a 24 h exposure to flucloxacillin at MIC and 20 mg/L did not reach statistical significance (-30.3%, *p* = 0.79 and −69.9%, *p* = 0.05 respectively). The incubation with a 20 mg/L concentration of flucloxacillin caused a significant biomass reduction for biofilms of strain 611 (−24.1%, *p* = 0.04). Fluorescence microscopy maximum intensity projection images of the Z-stacks at 20x magnification are shown in Figure 2C for strain ATCC 33591 and in Figure 3C for strain ATCC 25923. ATCC 33591 control biofilms uniformly covered the surface of the coupons. Live (green) cells were the most prevalent, but a small proportion of dead (red) cells was observed. Re-incubated controls were comparable without or with vancomycin. ATCC 25923 control biofilms appeared to have a looser aspect than those of ATCC 33591. The proportion of dead cells remained stable after re-incubation without flucloxacillin but appeared to increase when the samples were reincubated with flucloxacillin at MIC or 20 mg/L.

### Antimicrobial Effects of Pulsed Lavage With and Without Sequential Antibiotics

The use of pulsed lavage significantly reduced the CFU counts in all MRSA and MSSA strains by 2.74 to 4.04 log_10_ when compared to T0 controls (Figures 2A, 3A). The remaining bacterial load was found to be sufficient to promote the regrowth of bacteria within the biofilms to baseline (T0) levels after 24 h. The addition of vancomycin at MIC inhibited the regrowth of the biofilms of strains ATCC 33591 and 749 and reduced the CFU counts of strain 676 (−1.29log_10_). Flucloxacillin at MIC inhibited the regrowth of strains 578 and 611 and reduced the CFU counts of strain ATCC 25923 (−2.11log_10_). Sequential treatment with pulsed lavage and then 24 h exposure to either vancomycin (MRSA strains) or flucloxacillin (MSSA strains) at a 20 mg/L concentration reduced the CFU counts in all strains by 1.90 to 2.54log_10_ when compared to coupons analyzed after pulsed lavage alone. The two-way ANOVA revealed a highly significant (*p* < 0.001) interaction parameters for all strains when considering the exposure to pulsed lavage and the reincubation of the samples as factors (Supplementary Data Tables 1, 2). These significant interaction parameters indicate a synergy of pulsed lavage and antibiotic therapy on CFU counts.

Pulsed lavage significantly reduced the biomass in all strains of MRSA and MSSA by 81.7% to 98% (Figures 2B, 3B). As was observed for CFU counts, the remaining bacteria restored the biofilms to control levels after a 24 h reincubation in medium. The successive exposure to pulsed lavage and vancomycin or flucloxacillin at either MIC or 20 mg/L inhibited the restoration of the biofilms to control levels. No subsequent reduction in biomass following exposure to antibiotics was observed. Comparably to CFU counts, the two-way ANOVA analysis showed a highly significant *(p* = 0.005 to *p* < 0.001) interaction parameter for all strains when considering the exposure to pulsed lavage and the reincubation of the samples as factors (Supplementary Data Tables 3, 4). Likewise, these results point toward a synergy of pulsed lavage and antibiotic therapy on biomass.

Microscopy was used to evaluate pulsed lavage samples (Figures 2C, 3C). Pulsed lavage removed most of the cells for both strains, leaving small clusters on the surface of the coupons. The remaining cells appeared to be mostly viable. As was observed for CFU counts and biomass measurements, the incubation of samples treated with pulsed lavage without antibiotics resulted in a complete restoration of the biofilms, with images similar to those of the controls (Figures 2C, 3C). The incubation with vancomycin or flucloxacillin at MIC or 20 mg/L did not alter the density of cell clusters of the samples. The proportion of dead cells seemed to increase when the samples were exposed to a therapeutic concentration of antibiotics when compared to controls.

## Discussion

Our results show a synergistic effect of sequential pulsed-lavage and antimicrobial therapy with either vancomycin or flucloxacillin at clinically relevant concentrations against *Staphylococcus aureus* biofilms grown on titanium coupons. This combination of pulsed lavage with antibiotics at concentrations compatible with a parenteral administration to simulate PJI has not been previously reported. Knecht et al. published on the combination of pulsed lavage and incubation with tobramycin- and vancomycin-loaded calcium sulfate beads, showing a strong synergy (20). However, the antibiotic concentrations eluted in the culture medium were not determined, limiting the extrapolation of the results. Wolcott et al. (27) studied the combination of pulsed lavage and gentamicin against *S. aureus* biofilms in a chronic wound model. A synergy of the two treatments was observed, but the concentration of gentamicin was far above the human C_max_ after the administration of a conventional dose, limiting the extrapolation of the results to a clinical setting. Collectively, prior data and the data herein underline the importance of surgical irrigation of infected wound and implant surfaces prior to the administration of adequate antibiotic doses to observe a strong synergy and achieve maximal sustainable reduction in bacterial inoculum.

The independent use of pulsed lavage against *S. aureus* biofilms appeared to remove most of the biofilm cells and biomass in our experiments. This contrasts with previous studies that reported a 1 to 2 log_10_ reduction in cell numbers following pulsed lavage (17, 18, 20). This discrepancy between our results and previous studies may be due to differences in strains, material surfaces or culture conditions. However, the conditions used here likely closer simulate clinical conditions, with a comparatively short distance between the nozzle and the coupons, and a volume of fluid to sample surface ratio of 40 mL/cm^2^. Moreover, we studied a larger variety of strains, limiting the confounding factor of strain-dependent effects.

Despite a substantial reduction in CFU counts, we noted that residual bacteria on the coupons after pulsed lavage were sufficient to restore a biofilm after a 24 h incubation, consistent with what was previously shown by other authors (18).

We observed that vancomycin at its recommended serum trough concentration had no effect on reducing MRSA bacterial inocula via CFU or biomass if the biofilms were not first disrupted by pulsed lavage, consistent with previous data that vancomycin activity within biofilms is poor. Several authors have described that vastly supratherapeutic concentrations of vancomycin were required to observe a significant reduction in CFU counts (28) or in the metabolic activity (23, 29) of MRSA biofilms. The low penetration of vancomycin in *S. aureus* biofilms may explain these observations (30).

In contrast, a limited strain-dependent effect of flucloxacillin was observed against MSSA biofilms in the absence of pulse lavage. Flucloxacillin is a narrow spectrum β-lactam antibiotic, directed against *Staphylococci* and *Streptococci*, which is recommended in combination with rifampicin for MSSA and methicillin-susceptible *S. epidermidis* prosthetic joint infections, alongside nafcillin and oxacillin (31–33). Only a few conflicting studies have been published about the *in vitro* effect of flucloxacillin against *S. aureus* biofilms, pointing toward a variable, strain-dependent effect (34–37), analogous to our observations.

Our study presents several limitations. First, biofilms were grown only on Ti6Al4V as a substrate. This decision was based on the previous observation by Urish et al. (17) that the differences between metallic substrates are tenuous when considering the effect of pulsed lavage. Second, we limited the growth period of the biofilms before treatment to 24 h. While the biofilms were mature from a microbiological perspective, it could be argued that older biofilms would develop a more complex structure that could change the effect of the treatments we used. Third, we used antibiotics at set concentrations. While vancomycin is often administered in a continuous infusion, flucloxacillin is usually administered on a 2 g, 4 times per day regimen and important variations in serum concentrations are observed over time between 2 administrations. We decided to use, in addition to the MIC, a concentration equivalent to the one observed 3 h after administration of a 2 g dose in order to mitigate this limitation. However, as this concentration remained constantly above the MIC for 24 h, the observed effects of flucloxacillin may be overestimated.

## Conclusion

A synergy of pulsed lavage and vancomycin or flucloxacillin was observed against *S. aureus* biofilms grown on titanium alloy coupons. This effect was never reported when considering clinically relevant antibiotic concentrations. These results confirm the need for thorough irrigation of the metallic surfaces of implants during DAIR procedures to facilitate the subsequent action of antibiotics.

## Data Availability Statement

The datasets generated for this study are available on request to the corresponding author.

## Author Contributions

HP, AR-S, OC, and FVB contributed to the conception and design of the study. GS and HR-V provided clinical strains for the study. HP carried out experiments and analyzed data. HP and FVB wrote sections of the manuscript. All authors contributed to manuscript revision, read, and approved the submitted version.

## Conflict of Interest

The Interpulse pulsed-lavage devices used for the experiments were gifted by Stryker Co., Kalamazoo, MI, USA. The authors declare that the research was conducted in the absence of any other commercial or financial relationships that could be construed as a potential conflict of interest.
