# Supplementary material for: Synergistic Effects of Pulsed Lavage and Antimicrobial Therapy Against Staphylococcus aureus Biofilms in an in-vitro Model
Source: Front Med (Lausanne). 2020 Sep 17;7:527. doi: 10.3389/fmed.2020.00527 (PMC7527469; doi:10.3389/fmed.2020.00527)
Supplement: Supplementary file 1 [file Data_Sheet_1.docx]

Supplementary data

Table 1

| *MRSA strains: Two-way ANOVA on CFU counts* | | | | | | |  |
| --- | --- | --- | --- | --- | --- | --- | --- |
| Strains | Source | DF | SS | MS | F | P value | |
| ATCC 33591 | Reincubation | 3 | 40.79 | 13.6 | F (3, 24) = 52.64 | P<0.001 | |
|  | Pulsed-Lavage | 1 | 84.16 | 84.16 | F (1, 24) = 325.8 | P<0.001 | |
|  | Interaction | 3 | 38.59 | 12.86 | F (3, 24) = 49.81 | P<0.001 | |
|  | Residuals | 24 | 6.199 | 0.2583 |  |  | |
|  | Total | 31 | 169.7 |  |  |  | |
| 676 | Reincubation | 3 | 25.13 | 8.378 | F (3, 24) = 54.87 | P<0.001 | |
|  | Pulsed-Lavage | 1 | 79.84 | 79.84 | F (1, 24) = 522.9 | P<0.001 | |
|  | Interaction | 3 | 31.14 | 10.38 | F (3, 24) = 67.98 | P<0.001 | |
|  | Residuals | 24 | 3.664 | 0.1527 |  |  | |
|  | Total | 31 | 139.8 |  |  |  | |
| 749 | Reincubation | 3 | 28.56 | 9.522 | F (3, 24) = 43.72 | P<0.001 | |
|  | Pulsed-Lavage | 1 | 74.2 | 74.2 | F (1, 24) = 340.7 | P<0.001 | |
|  | Interaction | 3 | 25.43 | 8.478 | F (3, 24) = 38.92 | P<0.001 | |
|  | Residuals | 24 | 5.227 | 0.2178 |  |  | |
|  | Total | 31 | 133.4 |  |  |  | |
| DF : degrees of freedom ; SS : sum of squares ; MS : mean square | | | | | | |  |

Table 2

| *MSSA strains: Two-way ANOVA on CFU counts* | | | | | | | | | | |  |
| --- | --- | --- | --- | --- | --- | --- | --- | --- | --- | --- | --- |
| Strains | Source | DF | | SS | | MS | | F | | P value | |
| ATCC 25923 | Reincubation | 3 | 104.7 | | 34.91 | | F (3, 24) = 60.24 | | P<0.001 | |  |
|  | Pulsed-Lavage | 1 | 48.77 | | 48.77 | | F (1, 24) = 84.15 | | P<0.001 | |  |
|  | Interaction | 3 | 24.46 | | 8.155 | | F (3, 24) = 14.07 | | P<0.001 | |  |
|  | Residuals | 24 | 13.91 | | 0.5796 | |  | |  | |  |
|  | Total | 31 | 191.9 | |  | |  | |  | |  |
| 578 | Reincubation | 3 | 49.93 | | 16.64 | | F (3, 24) = 42.52 | | P<0.001 | |  |
|  | Pulsed-Lavage | 1 | 81.59 | | 81.59 | | F (1, 24) = 208.5 | | P<0.001 | |  |
|  | Interaction | 3 | 28.24 | | 9.413 | | F (3, 24) = 24.05 | | P<0.001 | |  |
|  | Residuals | 24 | 9.394 | | 0.3914 | |  | |  | |  |
|  | Total | 31 | 169.2 | |  | |  | |  | |  |
| 611 | Reincubation | 3 | 73.75 | | 24.58 | | F (3, 24) = 79.85 | | P<0.001 | |  |
|  | Pulsed-Lavage | 1 | 81.52 | | 81.52 | | F (1, 24) = 264.8 | | P<0.001 | |  |
|  | Interaction | 3 | 26.47 | | 8.825 | | F (3, 24) = 28.67 | | P<0.001 | |  |
|  | Residuals | 24 | 7.388 | | 0.3079 | |  | |  | |  |
|  | Total | 31 | 189.1 | |  | |  | |  | |  |
| DF : degrees of freedom ; SS : sum of squares ; MS : mean square | | | | | | | | | | |  |

Table 3

| *MRSA strains: Two-way ANOVA on biomass* | | | | | | |  |
| --- | --- | --- | --- | --- | --- | --- | --- |
| Strains | Source | DF | SS | MS | F | P value | |
| ATCC 33591 | Reincubation | 3 | 38282 | 12761 | F (3, 24) = 14.85 | P<0.001 | |
|  | Pulsed-Lavage | 1 | 33695 | 33695 | F (1, 24) = 39.21 | P<0.001 | |
|  | Interaction | 3 | 16838 | 5613 | F (3, 24) = 6.532 | P=0.002 | |
|  | Residuals | 24 | 20622 | 859.2 |  |  | |
|  | Total | 31 | 109437 |  |  |  | |
| 676 | Reincubation | 3 | 21349 | 7116 | F (3, 24) = 51.32 | P<0.001 | |
|  | Pulsed-Lavage | 1 | 38133 | 38133 | F (1, 24) = 275 | P<0.001 | |
|  | Interaction | 3 | 9099 | 3033 | F (3, 24) = 21.87 | P<0.001 | |
|  | Residuals | 24 | 3328 | 138.7 |  |  | |
|  | Total | 31 | 71909 |  |  |  | |
| 749 | Reincubation | 3 | 22114 | 7371 | F (3, 24) = 41.31 | P<0.001 | |
|  | Pulsed-Lavage | 1 | 37638 | 37638 | F (1, 24) = 210.9 | P<0.001 | |
|  | Interaction | 3 | 12613 | 4204 | F (3, 24) = 23.56 | P<0.001 | |
|  | Residuals | 24 | 4283 | 178.4 |  |  | |
|  | Total | 31 | 76649 |  |  |  | |
| DF : degrees of freedom ; SS : sum of squares ; MS : mean square | | | | | | |  |

Table 4

| *MSSA strains: Two-way ANOVA on biomass* | | | | | | | |
| --- | --- | --- | --- | --- | --- | --- | --- |
| Strains | Source | DF | SS | MS | F | P value |  |
| ATCC 25923 | Reincubation | 3 | 128267 | 42756 | F (3, 24) = 44.43 | P<0.001 |  |
|  | Pulsed-Lavage | 1 | 14610 | 14610 | F (1, 24) = 15.18 | P<0.001 |  |
|  | Interaction | 3 | 15854 | 5285 | F (3, 24) = 5.492 | P=0.005 |  |
|  | Residuals | 24 | 23093 | 962.2 |  |  |  |
|  | Total | 31 | 181825 |  |  |  |  |
| 578 | Reincubation | 3 | 22625 | 7542 | F (3, 24) = 138.1 | P<0.001 |  |
|  | Pulsed-Lavage | 1 | 50168 | 50168 | F (1, 24) = 918.5 | P<0.001 |  |
|  | Interaction | 3 | 6500 | 2167 | F (3, 24) = 39.67 | P<0.001 |  |
|  | Residuals | 24 | 1311 | 54.62 |  |  |  |
|  | Total | 31 | 80603 |  |  |  |  |
| 611 | Reincubation | 3 | 20168 | 6723 | F (3, 24) = 60.51 | P<0.001 |  |
|  | Pulsed-Lavage | 1 | 32823 | 32823 | F (1, 24) = 295.4 | P<0.001 |  |
|  | Interaction | 3 | 10358 | 3453 | F (3, 24) = 31.08 | P<0.001 |  |
|  | Residuals | 24 | 2667 | 111.1 |  |  |  |
|  | Total | 31 | 66016 |  |  |  |  |
| DF : degrees of freedom ; SS : sum of squares ; MS : mean square | | | | | | |  |

Figure 1


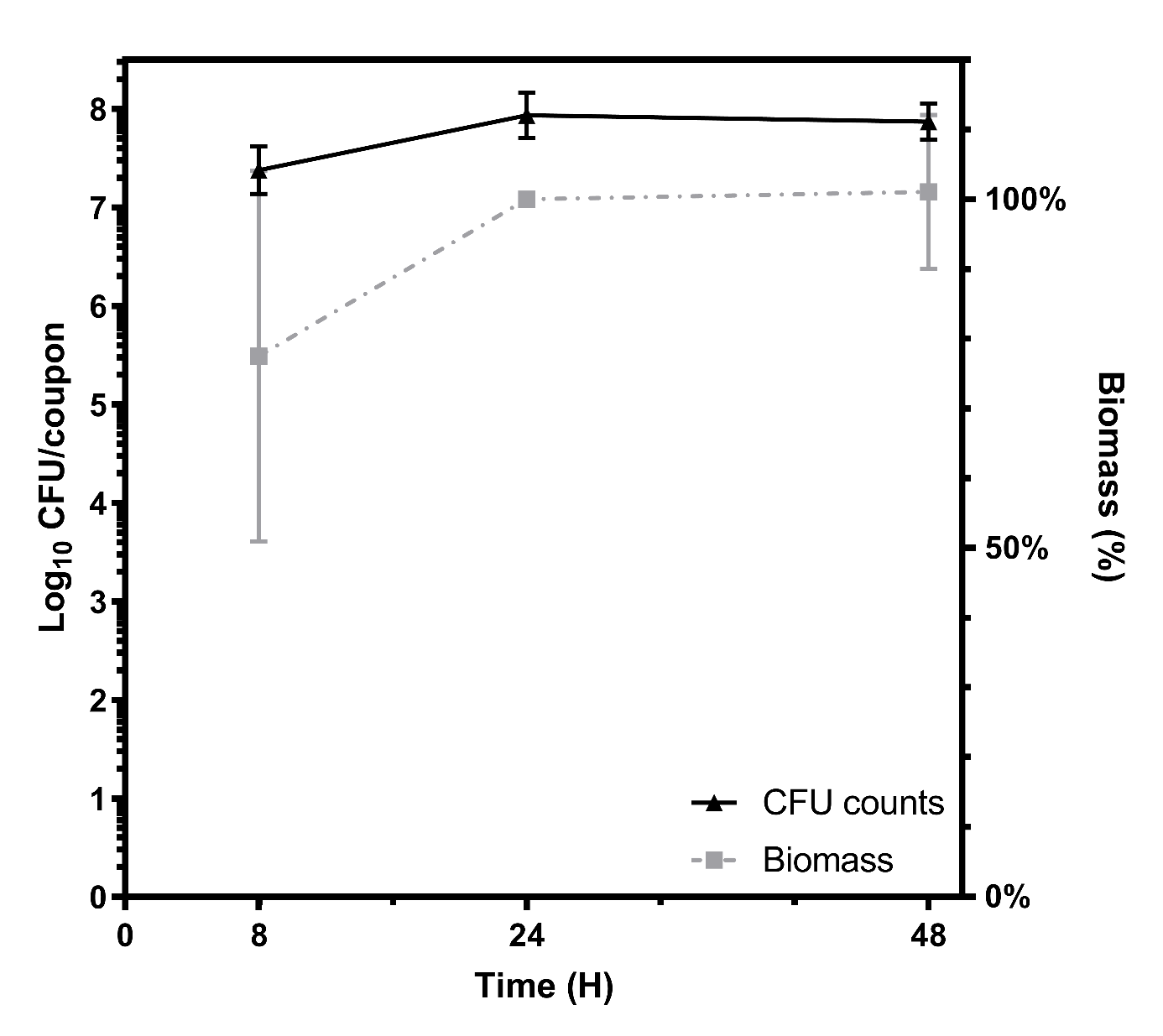


Growth of ATCC 33591 biofilms on Ti6Al4V coupons over time. CFU counts (triangles, black) expressed in logarithm (left axis), biomass (squares, grey), expressed in percentage of 24h biofilms biomass (right axis). Data expressed as means of 3 experiments and SEM.
